# Incorporation of Rubber Powder as Filler in a New Dry-Hybrid Technology: Rheological and 3D DEM Mastic Performances Evaluation

**DOI:** 10.3390/ma9100842

**Published:** 2016-10-18

**Authors:** Valeria Vignali, Francesco Mazzotta, Cesare Sangiorgi, Andrea Simone, Claudio Lantieri, Giulio Dondi

**Affiliations:** DICAM Department, University of Bologna, Viale Risorgimento 2, Bologna 40136, Italy; francesco.mazzotta2@unibo.it (F.M.); cesare.sangiorgi4@unibo.it (C.S.); andrea.simone@unibo.it (A.S.); claudio.lantieri2@unibo.it (C.L.); giulio.dondi@unibo.it (G.D.)

**Keywords:** rubber powder, limestone filler, mastics, Multiple Stress Creep Recovery, Frequency Sweep test, 3D discrete element modeling, burger viscoelastic model, Dynamic Shear Rheometer, complex modulus, phase angle

## Abstract

In recent years, the use of crumb rubber as modifier or additive within asphalt concretes has allowed obtaining mixtures able to bind high performances to recovery and reuse of discarded tires. To date, the common technologies that permit the reuse of rubber powder are the wet and dry ones. In this paper, a dry-hybrid technology for the production of Stone Mastic Asphalt mixtures is proposed. It allows the use of the rubber powder as filler, replacing part of the limestone one. Fillers are added and mixed with a high workability bitumen, modified with SBS (styrene-butadiene-styrene) polymer and paraffinic wax. The role of rubber powder and limestone filler within the bituminous mastic has been investigated through two different approaches. The first one is a rheological approach, which comprises a macro-scale laboratory analysis and a micro-scale DEM simulation. The second, instead, is a performance approach at high temperatures, which includes Multiple Stress Creep Recovery tests. The obtained results show that the rubber works as filler and it improves rheological characteristics of the polymer modified bitumen. In particular, it increases stiffness and elasticity at high temperatures and it reduces complex modulus at low temperatures.

## 1. Introduction

In order to minimize the environmental impacts of roads construction and maintenance, it is necessary to find solutions able to increase the pavements performance while reducing the externalities. In this context, the use of recycled materials, suitable for other re-use, must be developed and promoted [1,2,3,4,5].

The fields of application of crumb rubber in road constructions are various. The research studies that validate them are as numerous as the amount of positive results achieved in recycling this material. The use of recycled rubber from End of Life Tires (ELT) into asphalt concretes started back in the 1960s. The technology that has spread more is the so-called Asphalt Rubber (AR) one. AR binders are produced with the industrial process invented by Charles McDonald and that is at the basis of the wet process. This process starts with the mixing of ELT ground rubber with the bituminous binder, using mechanical stirring systems, working at temperatures ranging between 190 and 218 °C, for a time interval of 45 to 60 min [6]. 

Numerous studies have shown that the addition of crumb rubber to the virgin bitumen, by means of the AR technology, produces binders with enhanced rheological performances. It improves, in fact, resistance to rutting, fatigue cracking and thermal cracking. It allows, moreover, a reduction of the thickness of asphalt overlays and reflective cracking potential [7,8].

However, AR technology generates some disadvantages such as high production temperatures and long mixing reaction, with consequent energy consumption.

In order to reduce these deficiencies, a new wet process concept has been developed in the last few years. Oliveira et al. (2013) have shown that the combination of AR technology with Warm Mix Additives (WMA) improves the production conditions of asphalt mixtures. It reduces, in particular, the temperature at which the mixtures are produced and compacted, improving the personnel working conditions, with the incorporation of very limited amounts of additive [9,10,11,12]. 

Next to the wet technology, powdered rubber is used in asphalt mixtures with the dry technology. In this case, the rubber powder replaces a part of stone aggregates, modifying the resulting lytic skeleton and giving place to a limited interaction with the bituminous binder. The reaction between bitumen and crumb rubber can be considered negligible, because the mixtures are produced without any significant interaction time between bitumen and rubber [13]. Several studies indicate that with the dry technology is possible to produce “gap-graded” and “open-graded” mixtures. For their formulation, the same grading curve used for mixtures produced with the wet method may be introduced. Bitumen proportion must be determined taking into account that the rubber tends to absorb bitumen oils during the mixing, transport and placement [14]. 

In this paper, a dry-hybrid technology for the production of Stone Mastic Asphalt mixtures is proposed. It is called “Dry-Hybrid Technology” and it allows the use of the rubber powder as filler, replacing part of the limestone one. Fillers are mixed with a high workability bitumen, modified with SBS (styrene-butadiene-styrene) polymer and paraffinic wax. 

The idea is to manually add into the mixer the rubber powder, without significant alterations of the production process of traditional modified asphalt mixtures. In this way, the rubber can be considered as a filler and not an aggregate.

The aim of this technology is to improve both mixture mechanical and workability characteristics. The mix of elastic properties performed by crumb rubber powder and of the advantages given by modified bitumen and additives, in fact, can produce mix oxidation reduction and performance increasing at high temperatures [9].

In this way, moreover, it is possible to reduce costs and externalities produced by bituminous binders traditionally modified with recycled rubber. The mixing of bitumen and rubber at reduced temperatures permits to decrease environmental emissions and energetic costs. Recent research studies, moreover, have been demonstrated that trough this technique the workers are less susceptible to harmful substances produced during the mix compaction [15,16].

The proposed technology has been applied on Stone Mastic Asphalts (SMA) because they have a high stone content, which forms a gap-graded skeleton-like stone structure, filled with a high viscosity bituminous mastic of bitumen and filler. SMA, properly designed and produced, has excellent properties. The stone skeleton, with its high internal friction, gives excellent shear resistance. The mastic, voidless and binder rich, gives to the mix good durability, resistance to cracking and good stability at high temperatures. The surface texture is rougher than that of dense graded asphalt and it assures good skid resistance, proper light reflection, reduced water spray and lower traffic noise [17,18,19,20]. 

In this research study, two different approaches have been used:

- Rheological approach, which comprises a macro-scale laboratory analysis and a micro-scale simulation. In the first, Frequency Sweep tests (FS) have been implemented. In the second, a micromechanical modeling, able to predict the rheological behavior of mastics starting from the understanding of their internal interaction, has been used. Since Discrete particle Element Method (DEM) treats particles as distinct bodies that interact together at contact points, it can be a very useful tool [21,22,23,24,25]. The software Particle Flow Code (PFC) was used in this study [26]. The DEM simulation, in particular, has been used in order to evaluate the rubber effects and the interaction among limestone filler, rubber and bituminous matrix inside the mastics.
- Performance approach, which comprises Multiple Stress Creep Recovery (MSCR) tests, in order to evaluate the permanent deformation resistance of the mastics also in nonlinear analysis.

The obtained data permit validating the proposed “Dry-Hybrid Technology”.

## 2. Materials

### 2.1. Mastics Design

To evaluate the proposed “Dry-Hybrid Technology” for the production of Stone Mastic Asphalts, three different SMAs have been investigated (SMA, SMA1.2 and SMA0.7).

The first one (SMA) has been designed using standard proportion, according to the Italian technical specifications [27]. The other two, instead, have been designed in order to study the influence of rubber on the mix performance. In SMA1.2, in particular, the bitumen content has been increased, to improve the rubber percentage respect to the limestone filler one. In SMA0.7, instead, the bitumen content has been reduced, to evaluate the inferior limit of rubber amount in the mixture.

The percentages of bitumen, limestone filler and rubber on the weight of aggregate of the three SMAs are reported in Table 1.

Starting from the designed SMAs, according to their proportions, three corresponding mastics have been extracted (MasSMA, MasSMA1.2 and MasSMA0.7).

They were produced with a warm mixing process. Warm bitumen, modified with polymer and waxes, and two types of filler (limestone filler and crumb rubber) have been used. The bitumen, in particular, was prepared by adding paraffinic wax (2% in weight of bitumen) and SBS (5% in weight of bitumen) to a neat 70/100 pen binder. The wax, instead, was obtained from coal gasification using the Fisher–Tropsch process [9]. The wax, which has a melting point of 90 °C, was added at 130 °C. In order to avoid phase separation, the samples were mixed with a Silverson mixer L4R (Silverson Machines Ltd., Chesham, UK), for one minute. The characteristics of the base bitumen, provided from the producer, are shown in Table 2.

In order to be used as fillers, both limestone filler and crumb rubber have been characterized geometrically and volumetrically, respectively through a gradation analysis and their volumetric mass. Rigden Voids and Delta Ring and Ball were also performed according to EN standards. Table 3 shows the components characteristics.

The determination of limestone filler (*C_f_*) and rubber (*C_r_*) concentration in the mastic, based on mass, is given by:

(1) $$C_{f}=\frac{M_{f}}{M_{b}+M_{f}+M_{r}}\cdot 100$$

(2) $$C_{r}=\frac{M_{r}}{M_{b}+M_{f}+M_{r}}\cdot 100$$

where *M_f_* is the mass of limestone filler in the mastic, *M_r_* is the mass of rubber in the mastic and *M_b_* is the mass of bitumen in the mastic.

The determination of ratio of limestone filler (*R_f_*) and of rubber (*R_r_*) to bitumen, based on mass, is given by:

(3) $$R_{f}=\frac{M_{f}}{M_{b}}$$

(4) $$R_{r}=\frac{M_{r}}{M_{b}}$$

For each mastic, the comparison of limestone filler to bitumen (by mass) and rubber to bitumen (by mass) ratios is shown in Table 4. The requirement within Superpave system is a filler to bitumen ratios ranging between 0.6 and 1.2, based on mass. 

In this investigation, the sum of limestone filler to bitumen (*R_f_*) and rubber to bitumen (*R_r_*) ratios is between 0.73 and 0.77, excluding the mastic containing only limestone filler (MasSMA), for which the ratio of 1.36 exceeds the Superpave upper limit. 

### 2.2. Mastics Volumetric Analysis

The volumetric analysis has been conducted on the three mastics. The calculation of the compositional volume of bitumen, filler and rubber particle (*V_f_*, *V_r_*, and *V_b_*) has been obtained through the following equations:

(5) $$V_{f}=\frac{\frac{M_{f}}{S_{f}}}{\frac{M_{b}}{S_{b}}+\frac{M_{f}}{S_{f}}+\frac{M_{r}}{S_{r}}}\cdot 100$$

(6) $$V_{r}=\frac{\frac{M_{r}}{S_{r}}}{\frac{M_{b}}{S_{b}}+\frac{M_{f}}{S_{f}}+\frac{M_{r}}{S_{r}}}\cdot 100$$

(7) $$V_{b}=\frac{\frac{M_{b}}{S_{b}}}{\frac{M_{b}}{S_{b}}+\frac{M_{f}}{S_{f}}+\frac{M_{r}}{S_{r}}}\cdot 100$$

where *M_f_* is the mass of limestone filler in the mastic, *M_r_* is the mass of rubber in the mastic, *M_b_* is the mass of bitumen in the mastic, *S_f_* is the specific gravity of filler, *S_r_* is the specific gravity of rubber and *S_b_* is the specific gravity of bitumen. 

The results of the equations are reported in Table 5.

### 2.3. Mastics Production

The mastics were produced by adding the mass of limestone filler and fine crumb rubber to heated bitumen, at a temperature of 160 °C. The mix of the three components has been conducted with a traditional mixer for one minute. This procedure simulates what happens in the production phase of the industrial plant. The mixing procedures are detailed as follows:

- limestone filler was inserted into a 160 °C oven for 24 h, to ensure moisture free particle surfaces;
- the bitumen was stored in a 5 L tin, preheated 7 h into a 160 °C oven, to make bitumen homogeneous and ready to mix;
- the accurate amount of bitumen was poured into a 1 L tin, and the tin with the bitumen was placed on a hot plate and kept at 160 °C;
- the bitumen was mechanically stirred for 30 s;
- the accurate mass of the limestone filler and crumb rubber was slowly added, and the mixing process followed so that fillers were homogeneously dispersed in the bitumen; and
- the obtained mastic was poured in a silicon mold (2 mm high and 8 mm diameter) and stored at 5 °C before starting the test.

## 3. Mastics Rheological Behavior

The evaluation of mastics rheological behavior has been conducted through a rheological approach. It comprises a macro-scale laboratory analysis and a micro-scale simulation.

In the first, linear viscoelastic analysis using Frequency Sweep tests (FS) have been implemented.

In the second, a DEM micromechanical modeling, able to predict the rheological behavior of mastics starting from the understanding of their internal interaction, has been used. The DEM simulation, in particular, has been performed in order to examine in depth the rubber effects and the interaction among limestone filler, rubber and bituminous matrix inside the mastics. 

### 3.1. Macro-Scale Laboratory Analysis

According to EN 14770 [37], a dynamic mechanical analysis, using oscillatory tests, was performed on the three mastics. An Anton Paar MCR302 Dynamic Shear Rheometer (DSR) (Anton Paar S.r.l., Graz, Austria) was used. 

Numerous research studies have shown that there is a reproducibility of the rheological tests with DSR in the study of bituminous mastics, confirming that the principles behind the rheological analysis of bitumen are validated also for these complex materials [38,39,40,41].

The tests were conducted under controlled strain, and the strain amplitude was limited within the linear viscoelastic (LVE) response of the samples (Table 6). 

Data were obtained from frequency sweep tests between 0.01 and 10 Hz, conducted between 10 and 60 °C. The 8 mm measurement system with 2 mm gap was used in the entire temperature range. The rheological parameters obtained from frequency sweep tests were the complex shear modulus (G*) and phase angle (*δ*). 

G* has been evaluated as a function of the absolute values of the peak to-peak shear stress (*τ*_MAX_) and of the peak to-peak shear strain (*γ*_MAX_), using Equation (8) [42]:

(8) $$\left|G*\right|=\frac{\tau_{MAX}}{\gamma_{MAX}}$$

*δ* has been evaluated in terms of time lag (Δ*t*) between the shear stress and strain, as a function of the loading time (*t*) using Equation (9):

(9) $$\delta =\frac{\Delta t}{t}\cdot 360$$

Adopting the principle of Time–Temperature Superposition (TTS), the master curves of G* and *δ* were constructed at the reference temperature of 30 °C. 

To define the horizontal shift factor value (*a_t_*), the Williams–Landel–Ferry (WLF) theory has been used [38,39,40,41]. In this way, *a_t_* is a function of two constants (*C*_1_ and *C*_2_) and of two temperatures, the measurement one (T) and the reference one (*T_ref_*), according to Equation (10):

(10) $$\log a_{t}=\frac{C_{1}\cdot \left(T-T_{ref}\right)}{C_{2}\cdot \left(T-T_{ref}\right)}$$

**Figure 1:**
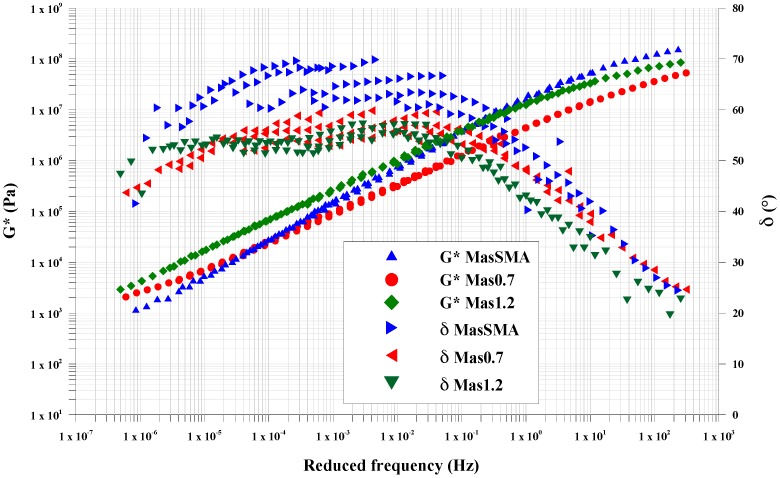
DSR master curves of tested mastics.

The master curves obtained in frequency sweep (FS) tests show differences in terms of values and trends, for both complex modulus (G*) and phase angle (*δ*) (Figure 1). 

The combined action of rubber and limestone filler increases the characteristics given by the SBS polymer to the bitumen. It reduces the mastics thermo-sensitivity and increases the viscoelasticity range.

In terms of master curve shape, all three materials exhibit the “s-shape” with two asymptotes (one for the high temperatures and one for low temperatures), typical of asphalt mixtures.

At low frequencies (LF), from 1 × 10^−7^ Hz to 1 × 10^−3^ Hz, Mas1.2 and Mas0.7 show complex modulus values higher than the mastic containing only limestone filler (MasSMA). At these frequencies, corresponding to high temperatures, the 14% and the 10% respectively of crumb rubber in weight on bitumen increase the complex shear modulus. Although the volumetric analysis shows that the filler presence is higher for MasSMA, the substitution of some limestone filler with fine crumb rubber increases the mastic response to permanent deformation at high temperatures. 

At medium frequencies (MF), from 1 × 10^−3^ Hz to 10 Hz, the G* of Mas1.2 and MasSMA tend to the same values, which means that they have the same shear stress response at medium temperatures between 20 and 40 °C.

The main differences can be observed at high frequencies (HF), from 10 Hz to 1 × 10^3^ Hz, where the mastic containing limestone filler (MasSMA) tends to the glassy modulus, showing G* between 1 × 10^8^ and 1 × 10^9^ Pa. At high frequencies, corresponding to low temperatures, the presence of rubber reduces the complex modulus and consequently the mastic fragile behavior.

In addition, in terms of phase angle, the trend is different for the studied mastics. At high frequencies, from 10 Hz to 1 × 10^3^ Hz, Mas0.7 and MasSMA have phase angle higher than Mas1.2, which has values between 20° and 40°. At medium frequencies, from 1 × 10^−3^ Hz to 10, Mas0.75 and Mas1.2 have *δ* values respectively between 50° and 55° and the MasSMA between 60° and 70°, showing the elastic response reduction without the crumb rubber. At low frequencies, from 1 × 10^−7^ to 1 × 10^−3^ Hz, the elastic response of the mastics containing crumb rubber is gradually exalted.

### 3.2. DEM Micro-Scale Simulation

#### 3.2.1. Introduction

The laboratory analysis showed that the interaction between fine crumb rubber and limestone filler provides the mastics with higher complex modulus and lower thermo-sensitivity and phase angles. In order to deeply investigate the influence of crumb rubber as a filler on the mastic behavior, a three-dimensional discrete element modeling approach has used.

As shown by numerous research works, in fact, it is possible to understand the microscale contacts mechanisms inside the material, from which its macroscale mechanical performances derive [21,43,44].

For each mastic, the discrete element simulation includes the following steps: definition of the model geometry, setting of contact material properties and simulation of the boundary and loading conditions of the frequency sweep test.

#### 3.2.2. Definition of the Model Geometry

According to Vignali et al. [42] the model geometry was defined using spherical particles, contained inside walls, which simulate the DSR device.

The bitumen spheres’ diameter was set to 200 µm, according to the magnitude of the bitumen film coating aggregates in bituminous mixes [45,46].

The limestone filler spheres’ diameter and the crumb rubber spheres’ diameter were set to 100 µm, according to grading curves (Table 3), in order to reduce calculation times. In fact, it should be noted that in DEM simulation it is almost impossible to take the fine particles fully into consideration, because not only this significantly increases the computational time, but it also affects the system’s capability to reach the equilibrium.

As measured by the manufacturer, the spheres’ density was set to 1040, 2560 and 1200 kg/m^3^ respectively for bitumen, limestone filler and crumb rubber. Density scaling technique was not used, despite it reducing computation time.

For each specimen, the spheres’ number was calculated following the volumetric proportion showed in Table 5.

The dynamic shear rheometer was simulated by three walls, one cylindrical and two planes. These last simulate the parallel plates that closed the cylinder at the top and at the bottom. The lower plane is fixed, while the upper one oscillates back and forth to create the shearing action. The cylinder has a contact stiffness equal to 10^2^ N/m, obtained from a calibration analysis [42].

**Figure 2:**
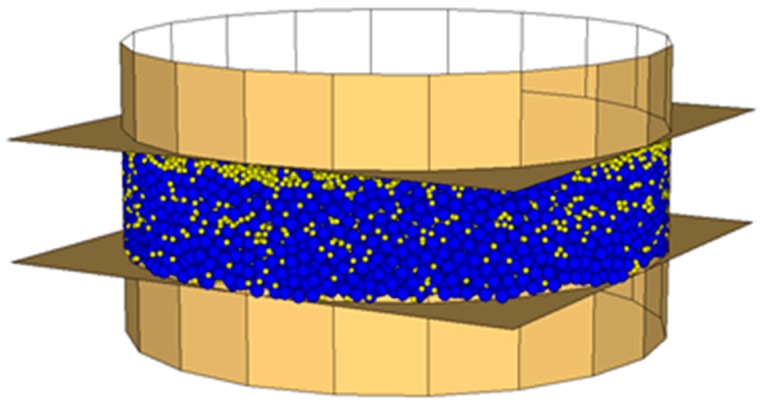
MasSMA, DEM simulation (bitumen particles in blue, limestone filler particles in yellow).

**Figure 3:**
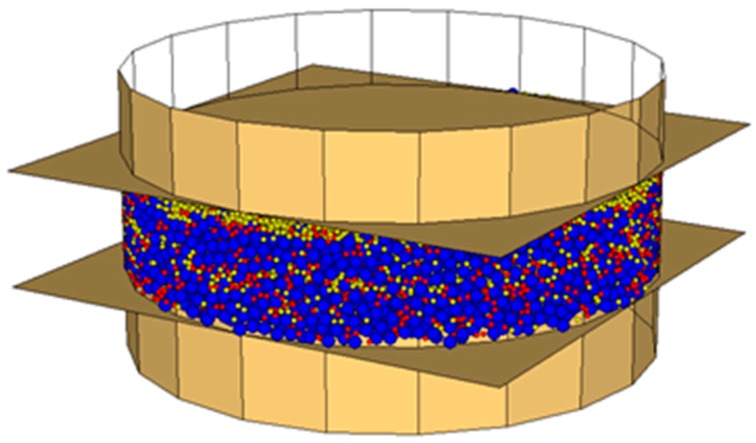
Mas0.7, DEM simulation (bitumen particles in blue, limestone filler particles in yellow, crumb rubber particles in red).

**Figure 4:**
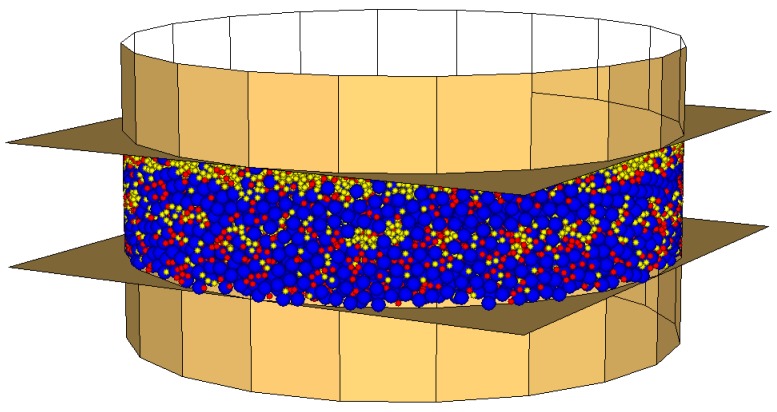
Mas1.2, DEM simulation (bitumen particles in blue, limestone filler particles in yellow, crumb rubber particles in red).

The sample of mastic, 2 mm thick and 8 mm in diameter, was generated inside the walls (Figure 2, Figure 3 and Figure 4).

#### 3.2.3. Description of the Contact Materials Parameters

According to Vignali et al. [42], for each mastic the thermal properties and the contact model was defined.

As indicated by the manufacturer, thermal properties were selected for each component of the mastic (bitumen, limestone filler and crumb rubber) in terms of (Table 7):

- specific heat at constant volume;
- coefficient of linear thermal expansion; and
- thermal resistance per unit length.

The contact model was defined choosing between two different approaches. In the existing literature, in fact, two different methods were used to build the DEM models with Burger’s constitutive relations [47]:

- in the first one, linear contact model was applied, in which the normal and shear stiffness of a discrete element chang
- es with loading time, based on the Burger’s constitutive relations [47,48]; and
- in the second, the embedded Burger’s model was applied, in which the viscoelastic properties of a contact are defined with the Burger’s constitutive relations and the model parameters are the stiffness or the viscosity at contact [49,50,51,52].

In this research study, the first approach was adopted.

It evaluates, in particular, the normal and shear stiffness of a discrete element by the following formulas [53,54]:

(11) $$k_{n}={\left[\frac{1}{K_{mn}}+\frac{t}{C_{mn}}+\frac{1}{K_{kn}}\cdot \left(1-e^{\frac{-t}{\tau_{n}}}\right)\right]}^{-1}$$

(12) $$k_{n}={\left[\frac{1}{K_{mn}}+\frac{t}{C_{mn}}+\frac{1}{K_{kn}}\cdot \left(1-e^{\frac{-t}{\tau_{n}}}\right)\right]}^{-1}$$

where: *t* is the loading time; *K_mn_* and *K_ms_* are the normal stiffness and the shear stiffness for Maxwell section, respectively; *C_mn_* and *C_ms_* are the normal viscosity and the shear viscosity for Maxwell section, respectively; *K_kn_* and *K_ks_* are the normal stiffness and the shear stiffness for Kelvin section, respectively; and *C_kn_* and *C_ks_* are the normal viscosity and the shear viscosity for Kelvin section, respectively. *τ_n_* and *τ_s_* are the normal and shear components of the relaxation time:

(13) $$\tau_{n}=\frac{C_{kn}}{K_{kn}}$$

(14) $$\tau_{s}=\frac{C_{ks}}{K_{ks}}$$

It was decided to equate the normal and shear direction parameters:

(15) $$K_{mn}=K_{ms}=K_{m}$$

(16) $$C_{mn}=C_{ms}=C_{m}$$

(17) $$K_{kn}=K_{ks}=K_{k}$$

(18) $$C_{kn}=C_{ks}=C_{k}$$

The response of the Burger model to a constant shear stress is characterized using the dynamic shear compliance and the dynamic shear modulus. These relationships are presented in following equations:

(19) $$\left|J^{*}\left(\omega \right)\right|=\sqrt{J\prime {\left(\omega \right)}^{2}+J\prime \prime {\left(\omega \right)}^{2}}$$

(20) $$\left|G^{*}\left(\omega \right)\right|=\frac{1}{\left|J*\left(\omega \right)\right|}=\frac{1}{\sqrt{J\prime {\left(\omega \right)}^{2}+J\prime \prime {\left(\omega \right)}^{2}}}$$

(21) $$J\prime \left(\omega \right)=\left(\frac{1}{K_{m}}+\frac{K_{k}}{{K^{2}}_{k}+\omega^{2}\cdot {C^{2}}_{k}}\right)$$

(22) $$J\prime \prime \left(\omega \right)=\left(\frac{1}{\omega \cdot C_{m}}+\frac{\omega \cdot C_{k}}{{K^{2}}_{k}+\omega^{2}\cdot {C^{2}}_{k}}\right)$$

(23) $$\left|G*\left(\omega \right)\right|=\frac{1}{\sqrt{{\left(\frac{1}{K_{m}}+\frac{K_{k}}{{K^{2}}_{k}+\omega^{2}\cdot {C^{2}}_{k}}\right)}^{2}+{\left(\frac{1}{\omega \cdot C_{m}}+\frac{\omega \cdot C_{k}}{{K^{2}}_{k}+\omega^{2}\cdot {C^{2}}_{k}}\right)}^{2}}}$$

where: $G^{\prime }\left(\omega \right)$ is the real part referred to as the storage shear modulus; $G^{\prime \prime }\left(\omega \right)$ is the imaginary part referred to as the loss shear modulus; $J^{\prime }\left(\omega \right)$ is the real part referred to as the storage shear compliance; and $J^{\prime \prime }\left(\omega \right)$ is the imaginary part referred to as the loss shear compliance.

Burger’s model parameters, in particular, were obtained by fitting to DSR measurements, according to the method developed by Baumgaertel and Winter [55]. The fitting procedure was based on minimizing an objective function corresponding to the sum of square of errors in predicting the storage and loss shear moduli over the available range of testing frequencies [48,53]:

(24) $$objective\_function=\sum_{j=1}^{m}\left[{\left(\frac{G\prime \left(\omega_{j}\right)}{G{\prime_{J}}^{0}}-1\right)}^{2}+{\left(\frac{G\prime \prime \left(\omega_{j}\right)}{G{{\prime \prime }_{J}}^{0}}-1\right)}^{2}\right]$$

where: *G*′*_j_*^0^ and *G*′′*_j_*^0^ are, respectively, the storage and loss shear moduli measured at the *j*th frequency *ω_j_*; *G*′*_j_* and *G*′′*_j_* are, respectively, the predicted storage and shear moduli; and *m* is the number of data points. The “Solver” option in Microsoft Excel was utilized for minimizing the objective function (Table 8).

#### 3.2.4. Boundary and Loading Conditions of the Frequency Sweep Test

According to Vignali et al. [42], an oscillatory shear load of constant amplitude was applied on the upper parallel wall, at several temperatures (between 10 and 60 °C) and different loading frequencies ranging between 0.01 and 10 Hz. Since in PFC forces can be applied only to balls and not to walls, an oscillatory shear angular velocity was used for each temperature. This velocity depends directly on the frequency amplitude, corresponding to the deformation of the linear viscoelastic range obtained in the amplitude sweep test.

#### 3.2.5. Obtained Results 

The results of numerical and laboratory tests have been analyzed, evaluating both the macro-scale and the particle-scale response.

##### Macro-Scale Results

Numerical results have been evaluated in terms of complex modulus (|G*|) and phase angle (*δ*). 

The first was calculated as a function of the maximum amplitude of the particles shear contact forces (*F_SMAX_*) and displacements (*S_SMAX_*), using Equation (25):

(25) $$\left|G*\right|=\frac{\left|F_{S_{MAX}}\right|}{\left|S_{S_{MAX}}\right|}$$

The second was evaluated as a function of the time lag between the peak shear contact force and the peak shear displacement (Δ*t*) and of the loading time of one cycle (*t*) using Equation (26):

(26) $$\delta =\frac{\Delta t}{t}\cdot 360$$

**Figure 5:**
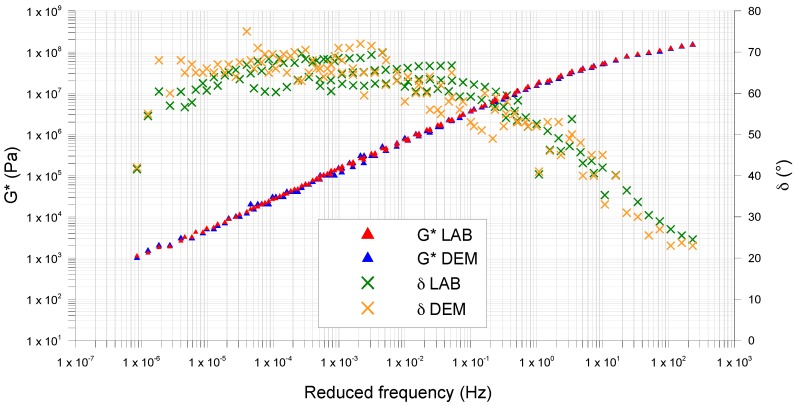
MasSMA, comparison between laboratory (LAB) and modeled (DEM) master curves.

**Figure 6:**
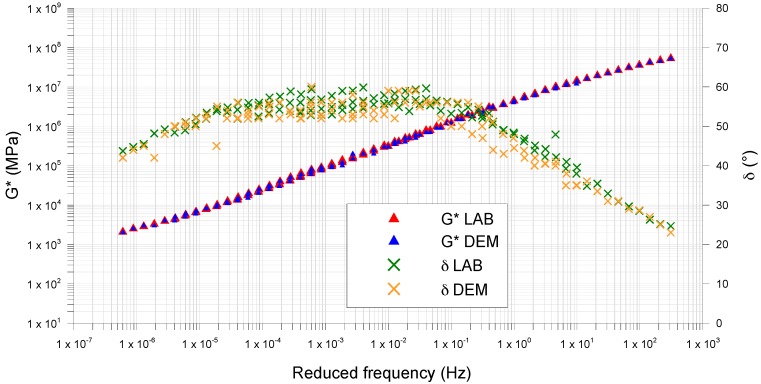
Mas0.7, comparison between laboratory (LAB) and modeled (DEM) master curves.

**Figure 7:**
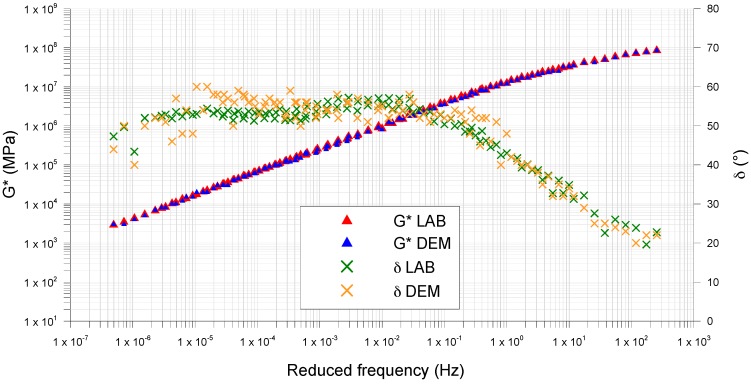
Mas1.2, comparison between laboratory (LAB) and modeled (DEM) master curves.

Figure 5, Figure 6 and Figure 7 compare the G* and *δ* master curves from the DEM simulation with the lab results, for each type of mastic. For all mastics, the measured data and the simulated ones have very similar trends, confirming that the DEM approach and assumptions used in this study, have good potential in predicting the mastic response in the frequency sweep configuration. 

**Figure 8:**
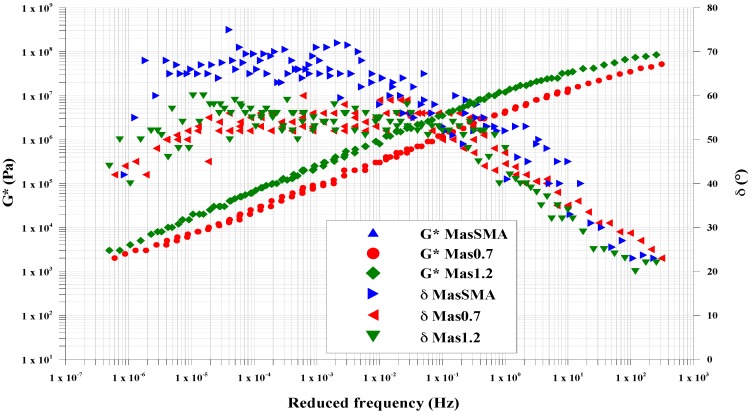
DEM master curves for all mastics.

In all tests, the complex modulus decreases and the phase angle increases with increasing temperature (Figure 8). In particular, the differences between the mastics containing fine crumb rubber and limestone filler (Mas0.7 and Mas1.2) and the MasSMA, containing only limestone filler, are consistent.

At high temperatures, Mas0.7 and Mas1.2 show a better response to shear stresses, attaining G* values higher than MasSMA. This tendency increases with the increase of crumb rubber content. 

At high frequencies, the crumb rubber properties reduce the mastic complex modulus and Mas1.2 appears less rigid than the MasSMA containing only limestone filler.

The master curves of phase angle also confirm this trend: at high temperatures, the elastic response of the mastic containing both rubber and limestone filler increases, exalting significantly the elastic behavior given by the polymer. At medium and low temperatures, the stress response of mastics Mas 0.7 and Mas1.2 shows phase angles lower than the ones of mastics containing limestone filler. 

Table 9 shows the mean error between DEM and lab data, for G* and *δ*. The obtained values are very small, confirming that the DEM approach used is effective in predicting the mastics response in the frequency sweep configuration.

The mean error is higher for the complex modulus than for the phase angle (Table 10). It always decreases with increasing of frequency, passing from low frequencies (LF) to medium ones (MF) and to high ones (HF). The complex modulus and phase angle, in all the range of frequency, the mean error decreases with the increasing of the crumb rubber content.

##### Micro-Scale Results

The particle-scale response was analyzed by considering both the contact forces between spheres and their displacements inside the specimens.

In order to evaluate the influence of crumb rubber as a filler on the mastic behavior, contact forces and particle displacements have been evaluated inside the modeled samples at the end of the frequency sweep test, at three different temperatures (10, 30 and 60 °C). Ten degrees Celsius is representative of low temperatures and high frequencies, at which crumb rubber content gives to the mix a better response to shear stresses. Sixty degrees Celsius is representative of high temperatures and low frequencies, at which the limestone filler have larger effects on the mastic. 

Due to the orientation of shear stresses, contact forces are mainly horizontally oriented. Shear contact forces have a uniform distribution inside the specimen and they are significantly larger than normal ones.

As the temperature increases, the maximum shear contact force inside the sample increases for each mastic (Table 11). The trend confirms the ability of crumb rubber, as a filler, to improve the sample complex modulus and the mastic resistance to permanent deformations at high temperatures.

The spheres’ displacements have been evaluated in a Cartesian coordinate system with:

- origin in the center of the upper horizontal plane of the model, which simulates the oscillating plate;
- z direction coincident to the vertical axis of symmetry of the sample, pointing to the lower plate.

For each mastic, from 10 to 60 °C, the total number of spheres displacements inside the sample increases and the 3D contacts network is characterized by a better interconnection between particles.

As shown in Table 12, for the same mastic, as the temperature increases, the maximum and minimum displacements of the particles in x, y and z directions increase. The z displacement component is always smaller than the x and y ones.

At high temperatures (low frequencies), MasSMA shows larger spheres displacements than the Mas0.7 and Mas1.2 ones, while at low temperatures (high frequencies) Mas1.2 shows the maximum displacements. At 10 °C, inside this sample with limestone and crumb rubber fillers, the particles move more independently of each other. Thus, the master curve trend, which shows that at high temperatures Mas1.2 is stiffer than MasSMA, is confirmed by the displacements of the spheres.

To better analyze the elastic behavior produced by crumb rubber, as a filler, on the mastic performance, the balls elastic displacements were evaluated.

These last were calculated on the horizontal plane of symmetry of the specimens (plane A-A), in order to discard the effect of the motion of parallel plates. 

The elastic displacements have been evaluated on the A-A plane, for each temperature (10, 30 and 60 °C), as the difference (∆) between the average displacement at the end of the test (loading frequency equal to 10 Hz) and the average one at the beginning of the test (loading frequency equal to 0.01 Hz). Hence, a small difference means that the average displacements at the beginning and at the end of the test are almost equal. Thus, it is an indicator of the material elasticity. 

As shown in Table 13, at each temperature, ∆ decreases from MasSMA, to Mas0.7 and to Mas1.2, confirming the elastic behavior produced by crumb rubber, as a filler, on the mastic performance.

## 4. Mastic Resistance Performance

The evaluation of the permanent deformation resistance of the mastics was conducted also in the nonlinear analysis, through a performance approach that comprises Multiple Stress Creep Recovery (MSCR) tests.

According to ASTM D7405 [56], these have been operated in the rotational mode at 58 °C, using 1 s creep load followed by 9 s recovery for each cycle. Ten creep and recovery cycles were run at 0.1 kPa creep stress, followed by ten at 3.2 kPa creep stress. For each cycle, two parameters were calculated: the percent recovery (%R) and the non-recoverable creep compliance (J_nr_).

**Figure 9:**
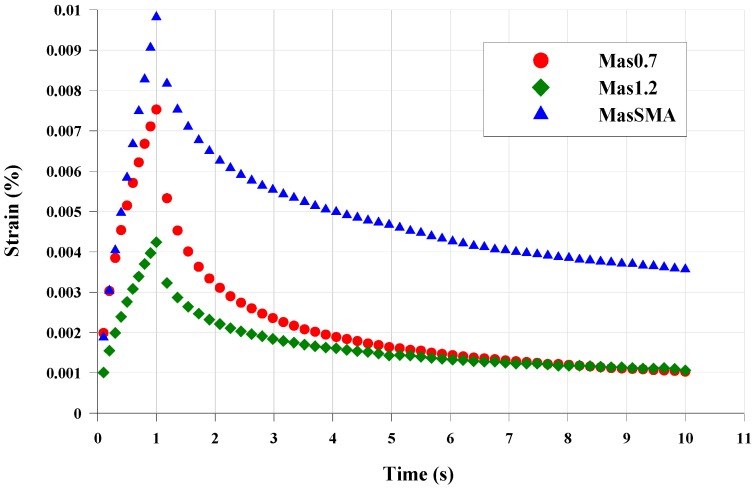
Shear strain vs. time, 1st cycle, 0.1 kPa, 58 °C.

Figure 9 illustrates the results of the first cycle at 0.1 kPa, at the test temperature of 58 °C. MasSMA accumulated the highest shear strain percentage. Mas1.2 and Mas0.7 show the same shear strain percentage. However the Mas1.2 reaches the minimum shear strain at the end of the 1 s shear stress application. 

**Figure 10:**
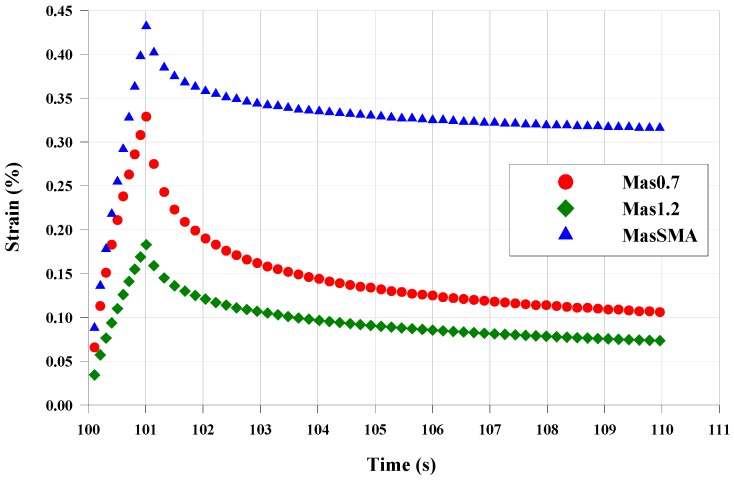
Shear strain vs. time, 1st cycle, 3.2 kPa, 58 °C.

Figure 10 illustrates the results of the first cycle at 3.2 kPa, at the test temperature of 58 °C. In addition, in this case, MasSMA accumulated the highest shear strain percentage. However, increasing the shear stress level, for the mastic containing both limestone filler and fine crumb rubber, the shear strain percentage is not the same, like the previous case. Mas1.2, containing more rubber, accumulates a lower deformation than the Mas0.7. 

**Figure 11:**
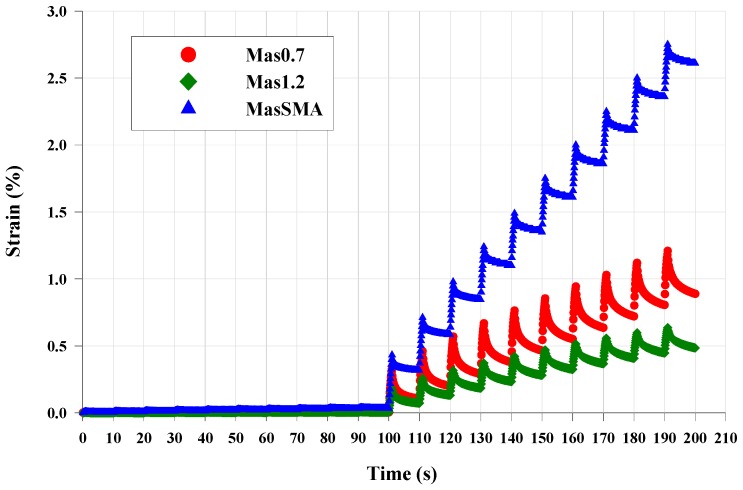
Shear strain vs. time, whole cycles, 58 °C.

Figure 11 shows that the rate of strain value significantly increases when the 3.2 kPa stress level starts. This effect goes up with crumb rubber reduction. Furthermore, the Mas1.2 always exhibits the stiffer behavior, accumulating less deformation at the end of the 10 cycles at 3.2 kPa, which the percentage of shear strain is roughly 0.5% at 58 °C. Moreover, the largest shear strain percentage corresponds to MasSMA. The final shear strain percentage for MasSMA at 58 °C is approximately 2%.

The phase angle reduction shows in FS tests at high temperatures is visible in terms of percent recovery in MSCR tests (Table 14). MasSMA highlights the lowest percentage of recovery at both shear stress levels. Mas1.2 has the highest recovery percentages at 0.1 and 3.2 kPa. For Mas0.7 the percentage of recovery, at both shear stress level, is between the values of mastic containing only limestone filler and the one with the highest percentage of crumb rubber. As described on linear analysis, this result confirms that the limestone filler and crumb rubber action increases both stiffness and elastic mastic response at the high temperatures. 

The creep compliance parameter (J_nr_) is a measure of the non-recoverable behavior of a binder caused by creep-recovery cycles. It is therefore suggested to describe the binder contribution to the asphalt mixture permanent deformations. In Table 15 are reported the J_nr_ values under 0.1 kPa and 3.2 kPa shear stresses, at the test temperatures of 58 °C. Mas1.2 has lower values of non-recoverable compliance at both shear stresses, showing a lower sensitivity to permanent deformations. In particular, at 3.2 kPa Mas1.2 illustrates a J_nr_ value of 0.02 1/kPa at 58 °C, while the J_nr_ values of MasSMA and Mas0.7 are 0.08 and 0.03 1/kPa at the same shear stress and temperature. The 14% in weight of fine crumb rubber acts on the bitumen and makes the mastic more elastic and less susceptible to non-recoverable deformations. Mas1.2 is potentially less exposed to the phenomenon of permanent deformation. MSCR tests confirm the frequency sweep results. At high temperatures the 14% in weight of crumb rubber increases the shear stress response of the mastic.

## 5. Conclusions

Based upon the developed research work, the following concluding remarks can be made:

- The presence of fine crumb rubber and limestone filler improves the mastic stiffness at high temperatures, increasing the mastic rutting resistance. The frequency sweep tests and the Multiple Stress Creep Recovery have shown that at high temperatures the mastic containing crumb rubber and limestone filler has higher complex modulus than the one with only limestone filler.
- The fine crumb rubber reduces the mastic thermo-sensitivity, because at low temperatures the mastic containing both rubber and limestone filler not tends to glassy modulus, increasing the mastic resistance to thermal cracking.
- The Discrete particle Element Method is a valuable and hopeful tool to study the rheological behavior of asphalt mastics.

The obtained results show that the proposed “Dry-Hybrid Technology” confirms the feasibility of mixing fine rubber powder with a high workability modified bitumen and aggregates. 

The obtained product is a modified bituminous mixture in which the rubber powder is mechanically added to the mixer, without significant alterations of the production process of a modified asphalt mixtures. The embedding to the mixer constitutes a continuity with the dry technique, which preserves the advantages in terms of absence of odors and of enhancement of the rheological properties of the binder.

The rubber operates more as an active filler than as aggregate. It changes the rheological properties of the asphalt mix, increasing the stiffness at high temperatures and reducing the complex modulus at low temperatures. 

The paraffinic wax in the bitumen, instead, can increase the workability of the modified binder inside of mixtures, whose volumetric proportioning of the components plays a key role the asphalt mixture elastic recovery increase.

The compatibility between powder and bituminous binder can be an advantage in the production of bitumen rich asphalts (SMA), for which the rubber can act as a stabilizer as the traditional fibers.

## Figures and Tables

**Table 1:** SMA components (%).

SMA	Bitumen	Limestone Filler	Crumb Rubber
SMA	6.60	9.00	0.00
SMA1.2	8.50	5.00	1.20
SMA0.7	7.50	5.00	0.75

**Table 2:** Properties of the warm modified bitumen (provided from the producer).

Property	Unit	Characteristic Value	Standard
Penetration @25 °C	dmm	25 ÷ 55	EN 1426 [28]
Softening Point	°C	70	EN 1427 [29]
Force Ductility test @10 °C	J/cm^3^	3	EN 13589 [30]
Dynamic Viscosity @160 °C	Pa·s	0.4 ÷ 0.7	EN 12596 [31]
Elastic Recovery @25 °C	%	80	EN 13398 [32]

**Table 3:** Percentages of bitumen, limestone filler and rubber on weight of bitumen.

Property	Unit	Limestone Filler	Crumb Rubber	Standard
Particle size range	mm	0 ÷ 0.063	0 ÷ 0.4	EN 933-10 [33]
Particle density	Mg/m^3^	2.73	1.01	EN 1097-7 [34]
Rigden voids	%	33.82	–	EN 1097-4 [35]
Δ Ring and Ball	°C	8	12	EN 13179-1 [36]

**Table 4:** Mastics composition.

Mastic	*C_f_* (%)	*C_r_* (%)	*R_f_*	*R_r_*
MasSMA	136	0	1.36	0.00
Mas1.2	59	14	0.59	0.14
Mas0.7	67	10	0.67	0.10

**Table 5:** Mastics volumetric composition.

Mastic	*V_f_* (%)	*V_r_* (%)	*V_b_* (%)
MasSMA	33	0	67
Mas1.2	16	11	73
Mas0.7	19	7	74

**Table 6:** Mastics Linear Visco-Elastic range.

Mastic	*γ* LVE (%)	Temperature (°C)
MasSMA	0.5	10
Mas1.2	0.8	10
Mas0.7	0.8	10

**Table 7:** Thermal properties.

Property	Bitumen	Limestone Filler	Crumb Rubber
Specific heat at constant volume (J/kg·°C)	1630	908	1737
Coefficient of linear thermal expansion (1/°C)	1.7 × 10^−5^	8 × 10^−6^	7 × 10^−5^
Thermal resistance per unit length (°C/W·m)	5.88	0.80	5.20

**Table 8:** Burger values.

Contact Model Parameter	“Solver” Value		
	MasSMA	Mas0.7	Mas1.2
Stiffness for Kelvin section (MPa)	0.13	0.10	0.19
Stiffness for Maxwell section (MPa)	22.67	5.51	13.90
Viscosity for Kelvin section (MPa·s)	6.97	2.10	7.72
Viscosity for Maxwell section (MPa·s)	44.50	37.46	144.59

**Table 9:** Mean error between LAB and DEM data for all mastics (%).

Property	G*	*δ*
MasSMA	6.67	4.45
Mas1.2	4.94	3.45
Mas0.7	5.93	4.04

**Table 10:** Mean error between LAB and DEM data for all mastics at difference frequency ranges (%).

Property	G*			*δ*		
	LF	MF	HF	LF	MF	HF
Mas1.2	6.15	5.66	3.20	4.00	3.41	3.10
Mas0.7	7.14	6.94	3.51	4.78	4.07	3.45
MasSMA	9.30	7.39	3.84	5.10	4.44	4.04

**Table 11:** Maximum shear contact force inside the specimen (10^−3^ N/m).

Temperature (°C)	10	30	60
MasSMA	2.809	6.173	11.357
Mas0.7	2.015	6.012	14.567
Mas1.2	2.477	8.138	17.323

**Table 12:** Maximum (sup) and minimum (inf) particles displacements in x (xdisp), y (ydisp) and z (zdisp) direction (mm).

Temperature (°C)	Mastic	xdisp inf	xdisp sup	ydisp inf	ydisp sup	zdisp inf	zdisp sup
10	MasSMA	0.000039	0.95	0.000038	1.10	0.000001	0.11
	Mas0.7	0.000041	1.29	0.000042	1.22	0.000001	0.14
	Mas1.2	0.000040	1.31	0.000040	1.29	0.000001	0.14
30	MasSMA	0.000049	1.50	0.000045	1.40	0.000003	0.30
	Mas0.7	0.000050	1.60	0.000048	1.70	0.000004	0.41
	Mas1.2	0.000043	1.40	0.000044	1.35	0.000002	0.20
60	MasSMA	0.000060	2.10	0.000056	2.20	0.000007	0.80
	Mas0.7	0.000055	1.80	0.000052	2.01	0.000006	0.67
	Mas1.2	0.000047	1.70	0.000045	1.80	0.000005	0.50

**Table 13:** Average displacement on the horizontal plane of symmetry of the specimens at the end of the test (Disp A-A 10 Hz) and at the beginning of the test (Disp A-A 0.01 Hz) (mm).

Temperature (°C)	Mastic	Disp A-A 0.01 Hz	Disp A-A 10 Hz	∆
10	MasSMA	0.295	0.580	0.285
	Mas0.7	0.560	0.796	0.131
	Mas1.2	0.665	0.595	0.035
30	MasSMA	0.950	1.060	0.225
	Mas0.7	0.835	1.195	0.115
	Mas1.2	1.080	0.980	0.030
60	MasSMA	1.160	1.475	0.470
	Mas0.7	1.145	1.265	0.120
	Mas1.2	1.005	1.180	0.020

**Table 14:** Percentage of recovery of MSCR test at 0.1 kPa and 3.2 kPa, at 58 °C (1/kPa).

Mastic	0.1 kPa	3.2 kPa
MasSMA	40	20
Mas1.2	87	32
Mas0.7	61	25

**Table 15:** Creep compliance parameter (J_nr_) of MSCR test at 0.1 kPa and 3.2 kPa, at 58 °C (1/kPa).

Mastic	0.1 kPa	3.2 kPa
MasSMA	0.03	0.08
Mas1.2	0.01	0.02
Mas0.7	0.01	0.03
